# Building blocks for enhancing personnel performance: activities, best practices and lessons learned from Ethiopia

**DOI:** 10.1186/2052-3211-7-S1-O2

**Published:** 2014-12-17

**Authors:** Daniel Taddesse, Suzanne Hoza, Tesfaye Seifu, Logan Cochrane

**Affiliations:** 1 Supply Chain Management System (SCMS), Addis Ababa, Ethiopia

## Background

The Supply Chain Management System (SCMS) program has worked in Ethiopia since 2006 to strengthen the public health supply chain. Increasing the performance and retention of personnel within the organization, and its governmental partners, has been, and continues to be, a priority. Five building blocks that are crucial for achieving these objectives, namely: engaging stakeholders, optimizing policies and plans, developing the workforce, increasing performance and professionalizing supply chain management, will be explored with practice-based case studies.

## Method

Each of the five building blocks (mentioned above) will be contextualized with the respective challenges being addressed. Following this, a concrete and practical example of an activity undertaken by SCMS Ethiopia in seeking to progressively increase performance and retain personnel. These examples will be drawn from SCMS activities internally and in supporting its primary governmental partner, the Pharmaceuticals Fund and Supply Agency (PFSA) of Ethiopia.

## Results

Participatory job description support, development, training, implementation and mentorship have supported the ownership of tasks by public sector supply chain professionals, clarified responsibilities and helped determine performance measures. Advocacy of the public health system has resulted in paradigm changes with decision makers. The development and adoption of curricula, and institutionalization of training within tertiary educational bodies throughout the country has demonstrated improved acceptance. This has resulted in an alignment of teaching practice with the expectations of organizations and government in the learning content and expected outcomes. The skills and knowledge of graduates has been strengthened, supporting the sector as a whole.

## Discussion

The experience of SCMS in Ethiopia provides a framework for organizations seeking to improve personnel performance. While the examples are specific, the challenges are common and lessons learned are applicable in a wide range of settings. This case study provide concrete examples of enhancing personnel performance in the public sector SCM in general and within a primary partner (PFSA) in particular. The long-term and strategic approach that was undertaken supported the achievements that SCMS was able to accomplish.

## Lessons learned

Participatory processes strengthen ownership and enhance adoption of change. Clear communication at each stage is essential to facilitate changes that are supported and advocated by the personnel involved. Improving performance requires long-term investment in human resource development, improved management strategies and advocacy for change beyond primary partners.

